# An innovative and collaborative partnership between patients with rare disease and industry-supported registries: the Global aHUS Registry

**DOI:** 10.1186/s13023-016-0537-5

**Published:** 2016-11-21

**Authors:** Len Woodward, Sally Johnson, Johan Vande Walle, Joran Beck, Christoph Gasteyger, Christoph Licht, Gema Ariceta, on behalf of the aHUS Registry SAB

**Affiliations:** 1 aHUS Alliance, Knutsford, Cheshire UK; 2 Department of Paediatric Nephrology, Great North Children’s Hospital, Newcastle upon Tyne, UK; 3 Department of Pediatric Nephrology, Safepedrug Consortium, Ghent University Hospital, Ghent, Belgium; 4 Ghent University, Ghent, Belgium; 5 Alexion Pharma GmbH, Zurich, Switzerland; 6 Division of Nephrology, The Hospital for Sick Children, Toronto, Canada; 7 Paediatric Nephrology, University Hospital Vall d’Hebron, Barcelona, Spain

**Keywords:** aHUS, Patient advocacy, Patient engagement, Registry

## Abstract

**Background:**

Patients are becoming increasingly involved in research which can promote innovation through novel ideas, support patient-centred actions, and facilitate drug development. For rare diseases, registries that collect data from patients can increase knowledge of the disease’s natural history, evaluate clinical therapies, monitor drug safety, and measure quality of care. The active participation of patients is expected to optimise rare-disease management and improve patient outcomes. However, few reports address the type and frequency of interactions involving patients, and what research input patient groups have. Here, we describe a collaboration between an international group of patient organisations advocating for patients with atypical haemolytic uraemic syndrome (aHUS), the aHUS Alliance, and an international aHUS patient registry (ClinicalTrials.gov NCT01522183).

**Results:**

The aHUS Registry Scientific Advisory Board (SAB) invited the aHUS Alliance to submit research ideas important to patients with aHUS. This resulted in 24 research suggestions from patients and patient organisations being presented to the SAB. The proposals were classified under seven categories, the most popular of which were understanding factors that cause disease manifestations and learning more about the clinical and psychological/social impact of living with the disease. Subsequently, aHUS Alliance members voted for up to five research priorities. The top priority was: “What are the outcomes of a transplant without eculizumab and what non-kidney damage is likely in patients with aHUS?”. This led directly to the initiation of an ongoing analysis of the data collected in the Registry on patients with kidney transplants.

**Conclusion:**

This collaboration resulted in several topics proposed by the aHUS Alliance being selected as priority activities for the aHUS Registry, with one new analysis already underway. A clear pathway was established for engagement between a patient advocacy group and an international research network. This should ensure the development of a long-term partnership which clearly benefits both groups.

## Background

In recent years, patients have become increasingly active in managing their own healthcare, in terms of both treatment decision-making processes and research participation. Thus, many patient organisations are actively engaging with clinical investigators and pharmaceutical companies. This new model has been shown to promote innovation, support patient-centred actions, and facilitate the development of new treatments [1]. While many patient advocacy groups may work closely with scientists and clinicians, there are few publications available describing the type of interactions that occur, how regularly they occur and what input to the research programme the groups have [2, 3].

### The problems of rare disease research

In the case of rare diseases, the development of effective treatment strategies can be hampered by a lack of knowledge of the condition. Issues include a lack of basic research on the natural history of the disease and difficulty in recruiting sufficient patients for effective clinical trials. Furthermore, the research that is performed may not answer the questions considered most important by patients [4]. Patient registries have been identified as tools to increase knowledge of the natural history of rare diseases, as well as providing a real-world setting in which clinical therapies, drug safety, and quality of care can be monitored [5]. Registries also provide a cohort of patients who may be suitable for enrolment into subsequent therapeutic trials. A global registry may also be the only way to collect sufficient data for robust statistical analyses. The active participation of patients will therefore lead to improved management of rare-diseases and patient outcomes [6].

Patient engagement in research can be interpreted in several ways, such as information sharing amongst rare disease groups and the use of patient-driven registries to facilitate data collection. However, such activities do not give patients input to the research process; for a successful registry, providing a voice for patients to help recruitment, encourage active participation and influence the research agenda is key. Although there may occasionally be reluctance on the part of patients and academics to engage in partnerships with industry, the significant effort and costs of a patient registry are best met through public/private partnership [7, 8]. Therefore enhancing communication and understanding between these groups is imperative.

### Atypical haemolytic uraemic syndrome

Atypical haemolytic uraemic syndrome (aHUS) is a rare genetic, life-threatening, chronic disease of children and adults caused by unregulated activation of the alternative complement pathway leading to systemic thrombotic microangiopathy (TMA) [9, 10]. A complement abnormality is described in 50–60% of patients but identification is not required for diagnosis [9, 11, 12]. Presentation can initially be mild, with patients reporting fatigue and influenza-like symptoms, or sudden and severe. aHUS often manifests as nonimmune microangiopathic haemolytic anaemia, thrombocytopenia, and acute organ failure. Historically, patients with aHUS had a poor prognosis with high morbidity due to chronic kidney disease, frequent graft failure after renal transplantation and high mortality rates [11, 12]. Differential diagnosis is based on exclusion of TMA caused by Shiga toxin-producing Escherichia coli-induced HUS and thrombotic thrombocytopenic purpura [9]. Other causes of TMA including (but not limited to) disorders of metabolism, drug toxicity and malignant hypertension should also be excluded [13]. Eculizumab, an anti-human C5 monoclonal antibody, is nowadays recommended for the treatment of aHUS following successful clinical trials in paediatric and adult patients with aHUS [14–16].

### The Global aHUS Registry

The Global aHUS Registry (US National Institutes of Health www.ClinicalTrials.gov Identifier NCT01522183) is an observational, non-interventional, multicentre registry of patients with aHUS, and is a product of a partnership between worldwide academia, patients with aHUS and Alexion Pharmaceuticals, Inc. The aims of the Registry are to assess the long-term patient outcomes, and collect and evaluate safety and effectiveness data specific to the use of eculizumab, fulfilling post-marketing regulatory requirements. The Global Registry is open to patients of all ages with a clinical diagnosis of aHUS; an identified complement gene mutation or factor H autoantibody is not required. Patients with secondary aHUS are eligible and physicians can record the potential precipitating cause. Patient data are collected following a research protocol and in accordance with International Conference on Harmonisation Good Clinical Practice Guidelines and the Declaration of Helsinki. Clinical and patient reported data (e.g. a fatigue score completed by the patient) are collected at study enrolment and every 6 months thereafter. The methodology of the aHUS Registry and characteristics of the first 516 patients enrolled (from 16 countries) have recently been published [17]. Amongst this patient cohort, 60% had received plasma therapy, 60% had a history of dialysis, and in 20% one or more kidney transplants had been performed. Overall, 59% of patients had received eculizumab.

Scientific oversight, governance and coordination of the Global aHUS Registry are provided by an independent scientific advisory board (SAB). The SAB includes members with expertise in key specialties related to management of aHUS (e.g. adult and paediatric nephrologists, haematologists, and/or transplant nephrologists/surgeons) as well as representation from Alexion Pharmaceuticals. Also, as a result of the engagement process described here, a patient representative is now included on the SAB. Data collected by the Global aHUS Registry are accessible to participating and nonparticipating physicians who may request data access or specific analyses by submitting a concept sheet via the aHUS Registry website (http://www.ahusregistry.com/) for evaluation by the SAB.

Here, we describe the feasibility of a new model of collaboration between an international group of patient organisations with a rare disease and an industry-supported disease registry, as a way to promote patient-centred research.

## Methods

### The Global Registry SAB

**Fig. 1:**
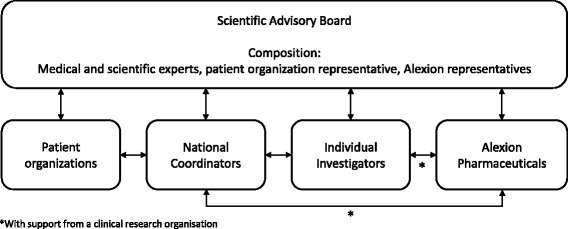
Stakeholder collaboration in the aHUS Global Registry. Patient organisations, individual investigators, national coordinators and Alexion Pharmaceuticals have direct representation on the Global Registry SAB. Individual investigators and national coordinators can also contact the sponsor, a process facilitated by the clinical research organisation responsible for the Registry

In order to improve registry management transparency and data analysis, the Global aHUS Registry SAB members discussed methods to promote patient benefit from participation and disease information sharing. Thus, in May 2014, the SAB members resolved to invite a representative from a patient organisation to join the SAB with the objective of strengthening the collaboration between academic experts and patient organisations in order to further understand patients’ needs and how the aHUS Registry could help address them. The relationships between the stakeholders involved in the Global Registry are shown in Fig. 1.

### The aHUS Alliance

In January 2013, representatives of patient organisations from six European countries advocating on behalf of aHUS patients met and agreed to collaborate as the aHUS Alliance (from Belgium, France, Italy, Russia, Spain and UK). Since then a further 6 organisations have become affiliated to the aHUS Alliance (from Australia, Canada, Germany, India, Netherlands and USA; http://www.ahusallianceaction.org/patient-associations/). One of the Alliance’s aims, listed in the inaugural “Declaration of Collaborative Aspirations”, was to work with international clinical aHUS research networks. In order to identify and prioritise areas of interest, the aHUS Alliance devised a questionnaire to conduct a global survey of patients with aHUS in 2014. The survey was available in English, French, Spanish, German, Italian and Dutch, and contained 28 questions seeking details of basic patient characteristics plus feedback on six issues commonly raised by patients with rare diseases; diagnosis, treatment, expert centres, research, registries and availability of information [18]. A similar survey was conducted in 2016, updated to include 46 questions [19]. The survey was promoted via social media and available on the Rare Connect and, in 2016, the aHUS Alliance websites.

Furthermore, following the approach by the aHUS Registry SAB about participating in its work, at its second General Meeting the Alliance elected a representative to sit on the Registry SAB. The primary roles of the patient advocate member of the SAB are described in Table 1.

**Table 1:** Main roles and commitments of the patient advocacy representative on the Global aHUS Registry SAB

Promoting interest in the Registry among patients with aHUS
• Generate programme awareness and interest within the patient community
• Assist with involvement of patient support groups and individual patients
• Provide information on the aHUS Registry to potential patients
• Provide advice and support to patients on aHUS Registry-related matters
Providing input to the SAB on patient priorities
• Act as the interface with other patient advocacy groups to provide a broad patient perspective from multiple countries to the Registry
• Inform the Registry of analyses and scientific questions of interest to the patient community
• Propose, discuss and evaluate programme objectives with the Registry SAB
• Provide ad hoc review of patient-related documents such as informed consent forms, patient leaflets and similar materials

*aHUS* atypical haemolytic uraemic syndrome, *SAB* scientific advisory board

To fulfil the aim of informing the SAB of research areas patients with aHUS considered the most important, the subject was included on the agenda for the third meeting of the aHUS Alliance in June 2015. Members were reminded about the call for research ideas to be brought to the meeting.

The session was facilitated by the Alliance’s aHUS registry representative. Initially there was a reluctance by the 22 representatives to respond, with an apprehension about articulating ideas in a way suitable for academics, disbelief that patient opinions would be considered seriously, and an unwillingness to give ideas to a commercial organisation for their perceived gain. To encourage contributions, it was highlighted that the Alliance would not critique, or assess in terms of the Registry data available, any proposals that were made.

A preliminary report was made available to all affiliates of the aHUS Alliance via an online file storage facility to allow other suggestions to be added. The final collated research suggestions were provided to the Chair of the aHUS Registry and reported to the SAB by the aHUS Alliance representative in November 2015 for consideration.

## Results

### aHUS Alliance global surveys

**Fig. 2:**
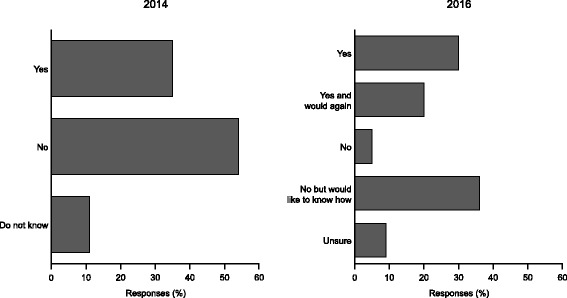
Involvement in aHUS research as reported by aHUS Alliance survey respondents in 2014 and 2016

Overall, responses were received from 214 patients or caregivers from 17 countries in 2014 and 233 respondents from 23 countries in 2016. Of those who responded, approximately a third reported they had participated in research (Fig. 2). In the 2016 survey, of the 96 responders who had not been involved in research, 85 (89%) said they would like to know how to participate.

**Fig. 3:**
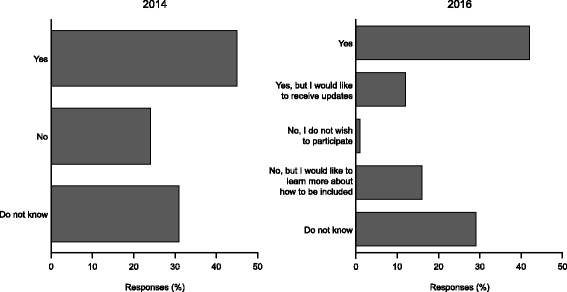
Enrolment in an aHUS patient registry as reported by aHUS Alliance survey respondents in 2014 and 2016

In the 2014 and 2016 surveys, a similar proportion reported being involved in any patient registry (45% and 42%, respectively), and in 2016 only 1% of respondents did not wish to participate (Fig. 3). Around 30% of responders in both surveys did not know if they or the person with aHUS they cared for were enrolled in a registry. Overall, there was a trend with time towards a more active and positive patient role in research.

**Fig. 4:**
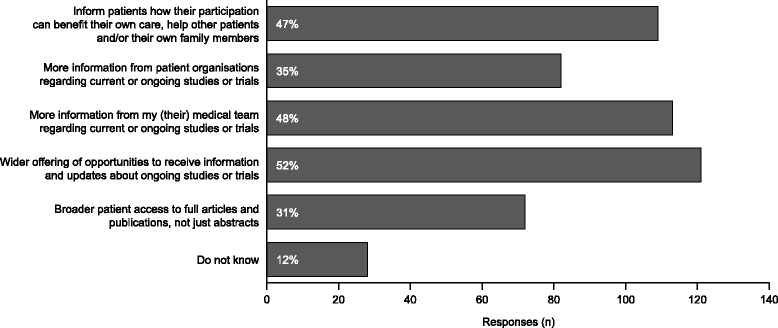
Responses to aHUS Alliance 2016 survey question on how to encourage patient participation in research. Patients were asked “In your opinion, what would encourage greater patient participation in research studies or clinical trials?”. Responses were fixed (no free text option) and more than one option could be selected. This question was only included in the 2016 survey

When asked what would encourage greater patient participation in research studies or clinical trials, three options were selected by approximately half of the respondents (Fig. 4). All the options, which covered receiving more information in one form or another, were selected by at least 30% of respondents.

### Classification and prioritisation of patient research proposals

In total, 24 ideas for research were generated. The ideas were then classified under seven categories (Table 2). Understanding the factors that cause a TMA manifestation provided the highest number of questions, followed by those around learning more about the clinical and psychological/social impact of living with the disease on patients and families. A wish to know about comparable aHUS patient characteristics provided the third highest number of topics. The categorised list was then presented to aHUS Alliance members and each country was requested to state its top five research priorities. The votes were well distributed among the 24 ideas revealing many topics were considered of interest. The highest scoring research topics are highlighted in bold (Table 2).

**Table 2:** Final questions and priority research areas from patients with aHUS and the aHUS Alliance

Topic	Question^a^	Score^b^
1. Disease onset	**• How transient is aHUS due to pregnancy and is there a role for prophylactic eculizumab in some cases?**	10
	**•** Does the incidence of aHUS vary in different environments e.g., urban, rural, coastal?	5
	**•** Are hormone changes during key life-stages a significant cause of aHUS onset?	5
	**•** Can boys with aHUS on-set at a young age grow out of it?	3
	**•** Do annual cycles in immune activity predict a time of year when aHUS onset is more likely?	2
	**•** Are those over 60 years-old with a genetic predisposition but no previous symptoms unlikely to develop aHUS or are they still at risk?	0
2. Diagnosis	**• What are the barriers to diagnosis, and how can they be overcome?**	15
	**• Can the degree of kidney function recovery be predicted by the time between aHUS onset and diagnosis/treatment?**	9
	**•** Is there a “golden period” for diagnosis which can predict more favourable outcomes for patients with aHUS?	8
3. Eculizumab treatment	**• Is it possible to ensure the effectiveness of eculizumab in the body?**	9
	**•** For how many days does eculizumab remain effective following administration and does it vary between patients?	0
4. Clinical effects	**• What are the outcomes of a transplant without eculizumab and what non-kidney damage is likely from any resulting aHUS onset?**	20
	**•** What is the incidence of (multi-organ) co-morbidities with aHUS for adults and children?	0
	**•** Are there differences between adults and children in terms of co-morbidities?	0
5. Psychological/social effects	**•** Is there any evidence as to whether not knowing the genetic cause or undergoing genetic testing causes the most anxiety?	4
	**•** What is the impact on the working life of adult patients with aHUS and carers of patients with aHUS?	0
	**•** What is the impact on education for children with aHUS?	0
	**•** What is the attitude of parents towards genetic screening of children?	0
	**•** What are the comparative self-esteem levels of patients with aHUS undergoing different treatment modalities?	0
	**•** To help family planning decision, can a risk matrix of the potential harm to a mother or child be developed?	0
6. Self-monitoring	**• Can a blood test be developed to allow patients in remission to monitor themselves?**	9
7. Patient differences	**•** What is the spectrum of the aHUS cohort in each country, and are there significant differences between them?	2
	**•** Is it possible to predict which patients will have the longest time in remission and which will be at the highest risk on new aHUS onset?	1
	**•** Does the spectrum of the aHUS cohort in each country change over time?	0
	**•** What is the frequency of my specific genetic predisposition in my country and other countries?	0

^a^Questions have been reproduced as formulated by the patient groups with only minor editing for clarity

^b^Countries comprising the aHUS Alliance were invited to vote for up to five research questions, which were scored as 5 points; most interesting, to 1 point; fifth most interesting. The sum of the scores received are reported. The top scoring questions are shown in bold

## Discussion

The effective engagement of patients in research on rare diseases is currently an area of increasing interest [8, 20]. This has come about as a result of governments giving greater priority and incentives for rare disease research, including through orphan drug legislation, [21, 22] and promotion by policy makers [20, 23]. This has occurred in parallel with a recent shift from a passive role for patients in research towards a desire to play a more involved role in the research process, as demonstrated by the survey outcomes. The lack of awareness of registry participation amongst respondents to the aHUS Alliance survey may be an area in which patient involvement in research can easily be improved. Patients may be unaware of the opportunity to enrol in a research registry if it is not offered by their physician. The fact that the Registry SAB initiated the collaboration by inviting a patient representative to join the team demonstrates that cooperation is bidirectional and reflects the recent change towards a new model of participation in research.

Amongst the research priorities identified, two diagnosis issues feature prominently. First, understanding the barriers to more rapid diagnosis and, second, how quickly diagnosis needs to be made to avoid irreversible organ damage. Understandably, a key concern of patients was around outcomes following kidney transplantation. This procedure was contraindicated in patients diagnosed with aHUS prior to the availability of eculizumab, and transplantation can also induce de novo aHUS. In some countries, aHUS patients on dialysis may be refused access to prophylactic eculizumab to prevent post-transplant recurrence of the disease and therefore this is a key issue for patient organisations around the world. However, data on this topic have been published in the medical literature, suggesting that the information available to patients with aHUS is poor. In this regard, patient organisations need to improve the dissemination of relevant information to patients and the participation of the aHUS Alliance in the Global Registry is an opportunity for patient organisations to inform and answer patient questions in a consistent and understandable way.

An ability to self-monitor aHUS symptoms also featured strongly, reflecting a desire of patients to feel in control of their condition. The issue of the time of year of aHUS onset has its roots in a casual discussion in the aHUS social media, but may be worthy of further study. Although not selected as a priority area of research, several members of the aHUS Alliance have an interest in this subject and a proposal to investigate this has been submitted to the SAB for consideration. Further, some of the questions raised demonstrate a potential lack of understanding of the disease and represent an opportunity for development of educational materials to address patient knowledge gaps.

Interestingly, no questions were raised regarding potential adverse effects of eculizumab, such as the risk of meningococcal infection, despite the primary purpose of the Registry being to determine the long-term safety of eculizumab treatment. This could be due a number of reasons, including physicians and patients considering the risk negligible, or the provision of patient information cards on treatment initiation and advice from patient organisations is sufficient explanation.

Following presentation of the report by the aHUS Alliance’s SAB representative, the research topics were given serious consideration for action. It was considered that a response to the aHUS Alliance’s list, including identification of knowledge gaps, would be a prerequisite to complete prioritisation. The subsequent work plan for the aHUS Registry in 2016 featured a number of topics proposed by the Alliance as priority activities. The aHUS Alliance had therefore not only been consulted but their response was considered and incorporated, creating a clear process of engagement for working with an international research network. This innovative way of generating research ideas has also been a learning process for the SAB members. For both parties to get the most from the collaboration, it is important to ensure the development of a long-term partnership.

### A paradigm shift for patient participation in disease-related research decision-making?

Patients with rare diseases have limited access to useful information to guide treatment decisions; providing patients with the opportunity to ask research questions may help to ensure that research efforts in rare diseases address relevant clinical questions and patient-centred health outcomes [20]. The active participation of patients and their representatives in research, whether a registry or clinical trial, can potentially lead to improvements in enrolment, data collection and quality, the credibility of results and their direct applicability to patients [24]. The inclusion of a patient representative on the Global aHUS Registry SAB will also improve the dissemination of outcomes to patients, thus increasing the transparency of the research process.

In order to promote changing the role of the patient in a registry from passive (*patient is a data point*) to active (*patient is a researcher*), [24] the participation of patients in the Global aHUS Registry is now actively supported by a patient representative working alongside the SAB. The patient advocate will assist in developing strategies to encourage voluntary patient participation in the Registry, will convey information from patients about their experience with the Registry, and will help the SAB share the results of the Registry with other patients.

### A formal structure for partnerships between patient groups and clinical researchers

The Dialogue Model [25] has been identified as a construct for the continuing development of the partnership in the context of the Registry. The Model has six stages (exploration, consultation, prioritisation, integration, programming and implementation), of which the emerging partnership has reached the start of the third stage. Not completing the early stages fully and satisfactorily can be predictive of a failure to sustain progress to successful implementation.

Completion of a robust systematic evidence assessment review of the knowledge gap needs to be reviewed by the SAB as well as the Alliance, to arrive at an agreed list of specific patient research priorities, which then must be integrated with the Registry’s other research agendas from clinicians and industry. With an integrated list of research topics finalised, it will then be possible to move to a sequential programming of topics for direct study by the SAB, or indirectly, through encouragement of external investigators to use the Registry data for other priority topics.

## Conclusions

The ongoing participation of patient advocacy groups in the activities of the Global aHUS Registry will help the Registry achieve its aims of establishing a robust database of patients with aHUS, characterising the long-term natural history of the disease, and enhancing the understanding of aHUS by publishing analyses of registry data. Engagement of patients with rare diseases in clinical research may promote research efforts directed to relevant clinical questions and patient-centred health outcomes.

## Abbreviations

aHUS: Atypical haemolytic uraemic syndrome
SAB: Scientific Advisory Board
TMA: Thrombotic microangiopathy
